# Eradication of hepatitis C virus is associated with the attenuation of steatosis as evaluated using a controlled attenuation parameter

**DOI:** 10.1038/s41598-018-26293-9

**Published:** 2018-05-18

**Authors:** Kohei Shimizu, Yoko Soroida, Masaya Sato, Hiromi Hikita, Tamaki Kobayashi, Momoe Endo, Mamiko Sato, Hiroaki Gotoh, Tomomi Iwai, Ryosuke Tateishi, Kazuhiko Koike, Yutaka Yatomi, Hitoshi Ikeda

**Affiliations:** 1 Department of Clinical Laboratory Medicine, The University of Tokyo, Tokyo, Japan; 2 Department of Gastroenterology, Graduate School of Medicine, The University of Tokyo, Tokyo, Japan

## Abstract

Chronic hepatitis C virus (HCV) infection was shown to cause hepatic steatosis or suppression of serum lipid levels. However, little is known about the changes in hepatic steatosis following HCV eradication. We aimed to evaluate this issue using the controlled attenuation parameter (CAP), which was recently shown to provide a standardized non-invasive measure of hepatic steatosis. We enrolled 70 patients with chronic HCV infections and steatosis (CAP of over 248 dB/m) who had achieved a sustained viral response at 12 weeks after discontinuation of antiviral treatment using direct-acting antivirals (DAA). We then evaluated the state of hepatic steatosis before and after HCV eradication. We also investigated the changes in serum parameters such as cholesterol and glucose levels. The median value of CAP level decreased significantly after HCV eradication from 273 dB/m to 265 dB/m (*P* = 0.034). Also, LDL and HDL cholesterol levels increased significantly after HCV eradication (*P* = 0.002 and *P* = 0.027, respectively). In conclusion, a decrease in hepatic steatosis after HCV eradication with DAA was revealed in chronic hepatitis C patients with significant steatosis. Cancellation of the viral effect is a possible underlying cause of hepatic steatosis improvement and increase in HDL and LDL cholesterol levels.

## Introduction

Hepatitis C virus (HCV) infection is known to cause chronic hepatitis, liver cirrhosis, and eventually hepatocellular carcinoma (HCC)^1^. Of note, about half of patients with chronic hepatitis caused by HCV have hepatic steatosis^2^, and the prevalence of hepatic steatosis increases up to as high as 75% in chronic hepatitis patients infected with HCV genotype 3a^2^, suggesting a close relationship between HCV and hepatic steatosis. Indeed, HCV core protein reportedly provides an anchor to the endoplasmic reticulum membrane of hepatocytes, allowing the core protein access to lipid droplets^3,4^, which could explain the close link between HCV and hepatic steatosis. Finally, the overexpression of the core protein alone is reportedly sufficient to cause hepatic steatosis in mice^5^. These lines of evidence suggest that HCV itself could cause steatosis in the liver.

On the other hand, a retrospective analysis of 3826 liver biopsies, among which 3021 biopsies were performed in HCV patients, revealed that the presence of hepatic steatosis in HCV patients was strongly associated with the features of metabolic syndrome^6^, suggesting that host factors, but not viral factors, may have important contributions to the mechanism of hepatic steatosis in HCV patients. To clarify the role of viral factors in the mechanism of hepatic steatosis, we sought to investigate the state of hepatic steatosis once HCV has been eradicated using recently established, direct-acting antivirals (DAAs).

To evaluate steatosis in the liver, a histological analysis of a liver biopsy specimen has been the golden standard, but the invasiveness of this procedure has long been a problem^2,3^. Abdominal ultrasonography is now commonly used to diagnose steatosis in the liver, but its use is unreliable for the evaluation of mild steatosis. Recently, the controlled attenuation parameter (CAP) has emerged as a reliable and noninvasive test to quantitatively evaluate steatosis. CAP is measured simultaneously with liver stiffness measurements (LSM) using transient elastography (TE) performed with a FibroScan^7^. A recent meta-analysis has revealed that CAP provides a standardized non-invasive measure of hepatic steatosis with an optimal cut-off of 248 for significant steatosis^8^. In the current study, CAP was used to evaluate hepatic steatosis.

## Patients and Method

### Patients

Six hundred and seventy-nine patients with chronic hepatitis C (CHC), who were treated with DAAs between March 2012 and June 2016 in the liver clinic at the University of Tokyo Hospital were enrolled. The inclusion criteria were as follows: (1) positivity for HCV RNA before treatment, (2) availability of abdominal ultrasonography, LSM and CAP measurements before and after treatment, (3) a CAP of more than 248 dB/m before treatment, and (4) the achievement of a sustained virological response (SVR) at 12 weeks after discontinuation of treatment.

The current study was performed in accordance with the ethical guidelines of the Declaration of Helsinki. Informed consent was obtained in the form of opt-out on the website. Patients who rejected to be enrolled in our study were excluded. The study design was included in a comprehensive protocol for retrospective studies at the University of Tokyo Hospital, and was approved by the University of Tokyo Medical Research Center Ethics Committee (approval number, 3683).

### Transient Elastography

CAP and LSM were measured using 3.5 MHz TE M probe (FibroScan 502 Touch; Echosens, Paris, France) after at least 4 hours of fasting. A TE evaluation had been performed within one month before the start of treatment. Data were used only when 10 successful measurements were obtained, the success rate was ≥60%, and the interquartile range was <30% of the median. According to a previously reported meta-analysis, the cut-off value for significant steatosis was set at 248 dB/m^8^.

#### Study endpoints

The primary endpoint of the current study was the change in hepatic steatosis measured using CAP before and after HCV eradication. The secondary endpoints were changes in serum parameters, such as the cholesterol and glucose levels, and the associations between the baseline characteristics and CAP changes induced by HCV eradication.

### Statistics

Data are shown as the median ± interquartile. To compare data obtained before and after treatment, a paired *t*-test was used for continuous variables and a chi-squared test was used to compare categorical data, where *P* < 0.05 was considered to reflect statistical significance. All the analyses were performed with the aid of SPSS software (ver. 19.0; IBM Corp., Armonk, NY, USA).

## Results

### Characteristics of patients

To examine the potential effect of HCV eradication on hepatic steatosis, we enrolled CHC patients with a CAP of over 248 dB/m, which was the optimal cut-off for significant hepatic steatosis reported in a recent meta-analysis^8^. The pre-treatment characteristics of the 70 patients who were ultimately enrolled are shown in Table 1. The median age of the patients was 66 years. The average observation period was 487 days. Regarding the regimen used for the treatment of HCV, sofosbvir/ledipasvir, daglatsvir/asnaprevir, ombitasvir/paritaprevir/ritonavir, elbasvir/grazoprevir, and sofosbuvir/ribavirin were used in 64.3% (45 patients), 12.9% (9 patients), 1.4% (1 patient), 1.4% (1 patient), and 20.0% (14 patients), respectively. Approximately 20% of all patients had received previous interferon based treatment. No patients had previous DAA treatment. The median CAP and LSM values were 273 dB/m and 7.3 kPa, respectively, before treatment. Fifty-six patients had genotype 1, and 14 patients had genotype 2.

**Table 1:** Patient characteristics.

Sex, n (%)	
Female	41 (58.6)
Male	29 (41.4)
Age (years)	66 (59–73)
Body mass index (BMI)	23.1 (21.2–26.0)
Genotype, n (%)	
type 1	56 (80.0)
type 2	14 (20.0)
Previous interferon based treatment, n (%)	
Present	16 (22.9)
Absent	54 (77.1)
DAAs, n (%)	
Sofosbvir/ledipasvir	45 (64.3)
daglatsvir/asnaprevir	9 (12.9)
ombitasvir/paritaprevir/ritonavir	1 (1.4)
elbasvir/grazoprevir	1 (1.4)
sofosbuvir/ribavirin	14 (20.0)
HCV RNA (LogIU/mL)	6.3 (5.7–6.6)
LSM (kPa)	7.6 (5.8–11.9)
CAP (dB/m)	273 (260–298)
AST (U/L)	43 (30–59)
ALT (U/L)	45 (27–62)
γ-GTP (U/L)	32 (19–51)
Platelet Count (×10^4^/μL)	18.4 (14.0–22.9)
Albumin (g/dL)	4.2 (4.0–4.4)
Triglycerides (mg/dL)	102 (81–165)
Total Cholesterol (mg/dL)	172 (150–207)
HDL Cholesterol (mg/dL)	52.9 (47.3–62.7)
LDL Cholesterol (mg/dL)	106.0 (81.0–134.5)
Fasting plasma glucose (mg/dL)	101 (94–124)
HbA1c (%)	5.8 (5.5–6.8)

*Data were expressed as the median values (1^st^–3^rd^ quartiles).

### Changes in CAP and LSM

**Figure 1:**
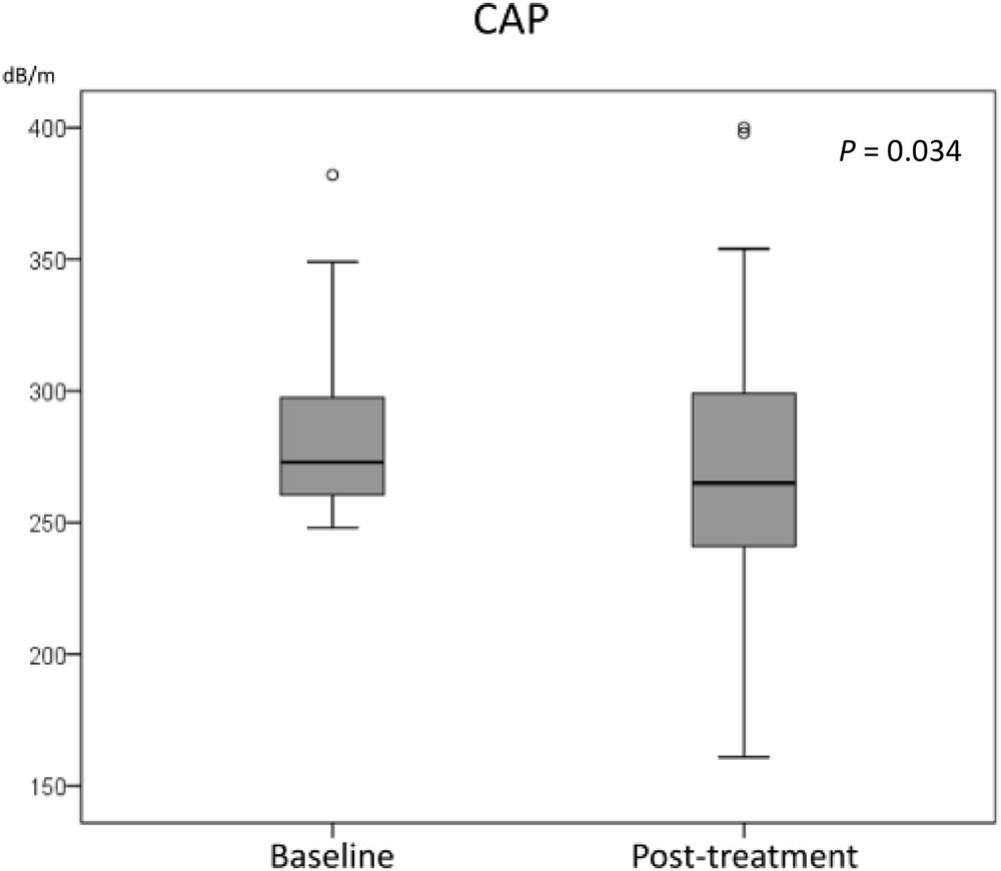
Alteration of CAP level before and after HCV eradication. The CAP level was significantly reduced after HCV eradication, from 282.7 dB/m to 268.3 dB/m (*P* = 0.034).

Figure 1 shows the change in CAP before and after treatment. The median value of CAP decreased after HCV eradication, from 273 dB/m to 265 dB/m (*P* = 0.034) during a median interval of 16.3 (12.6–18.5) months. A reduction in CAP after treatment was observed in 40 of the 70 patients. On the other hand, the LSM values before and after treatment were 7.6 kPa and 6.7 kPa, respectively, and significantly reduced after HCV eradication (*p* = 0.026). These results indicate that hepatic steatosis was attenuated with HCV eradication in CHC patients.

### Changes in clinical parameters

The changes in various clinical parameters before and after treatment are shown in Table 2. After treatment, the serum levels of AST and ALT were decreased, and the platelet count and serum levels of albumin, HDL cholesterol and LDL cholesterol were increased in accordance with the cure of chronic hepatitis C (CHC).

**Table 2:** Changes in clinical parameters after HCV eradication.

Parameters	Values		*P* - value
	Baseline	Post**-**treatment	
LSM (kPa)	7.6 (5.8–11.9)	6.7 (4.5–10.1)	0.026
CAP (dB/m)	273 (260–298)	265 (238–305)	0.034
AST (U/L)	43 (30–59)	22 (18–27)	<0.001
ALT (U/L)	45 (27–62)	17 (12–23)	<0.001
γ-GTP (U/L)	32 (19–51)	21 (16–27)	0.062
Platelet Count (×10^4^/μL)	18.4 (14.0–22.9)	20.0 (15.4–24.4)	0.001
Albumin (g/dL)	4.2 (4.0–4.4)	4.3 (4.1–4.4)	<0.001
Triglycerides (mg/dL)	102 (81–165)	116 (79–184)	0.078
Total Cholesterol (mg/dL)	172 (150–207)	190 (157–224)	0.21
HDL Cholesterol (mg/dL)	52.9 (47.3–62.7)	59.4 (49.4–83.2)	0.027
LDL Cholesterol (mg/dL)	106.0 (81.0–134.5)	109 (85–143)	0.002
Fasting plasma glucose (mg/dL)	101 (94–124)	107 (95–120)	0.079
HbA1c (%)	5.8 (5.5–6.8)	6.5 (5.7–6.9)	0.754

*Data were expressed as the median values (1^st^–3^rd^ quartiles).

### Baseline clinical parameters in patients with improved hepatic steatosis and in those with non-improved hepatic steatosis

We divided the enrolled patients into two groups**:** patients with improved hepatic steatosis, and those with non-improved hepatic steatosis after HCV eradication. Table 3 depicts the baseline clinical parameters of the patients with improved hepatic steatosis and of those with non-improved hepatic steatosis. Patient sex and presence or absence of previous interferon based treatment were not associated with steatosis change after HCV eradication (p = 0.83 and p = 0.94, respectively). Regarding viral factors, the viral load was not different between the two groups. In the patients with genotype 1, hepatic steatosis was improved in 31 out of 57 patients (54.4%), and in those with genotype 2, hepatic steatosis was improved in 9 out of 14 patients (64.3%). As host factors, HDL cholesterol was significantly higher and HbA1c was significantly lower in the patients with improved hepatic steatosis than in those with non-improved hepatic steatosis.

**Table 3:** Baseline clinical parameters in patients with and those without improved hepatic steatosis.

Parameters	Values		*P* - value
	Decreased (improved)	non-improved	
age	66 (58–75)	66 (60–71)	0.974
HCV RNA (LogIU/mL)	6.1 (5.6–6.6)	6.4 (6.2–6.6)	0.241
LSM (kPa)	8.0 (5.7–12.0)	7.5 (5.9–11.9)	0.462
CAP (dB/m)	280 (263–303)	264 (258–293)	0.197
AST (U/L)	41 (24–53)	45 (30–63)	0.386
ALT (U/L)	40 (24–53)	51 (30–68)	0.181
γ-GTP (U/L)	32 (17–52)	32 (22–51)	0.436
Platelet Count (×10^4^/μL)	18.9 (16.1–22.9)	17.0 (11.5–23.2)	0.084
Albumin (g/dL)	4.2 (4.0–4.4)	4.2 (4.0–4.4)	0.941
Total Cholesterol (mg/dL)	179 (154–208)	166 (145–178)	0.801
HDL Cholesterol (mg/dL)	53 (52–65)	50 (40–54)	<0.001
LDL Cholesterol (mg/dL)	110 (81–132)	101 (82–139)	0.644
HbA1c	5.7 (5.3–6.0)	6.8 (5.5–7.1)	0.033

*Data were expressed as the median values (1^st^–3^rd^ quartiles).

### Baseline clinical parameters in patients decreased LDL and HDL levels and in those with increased LDL and HDL levels

We also investigated baseline clinical parameters of patients with and without improved cholesterol (LDL and HDL) level after HCV eradication. Supplementary Table 1 depicts the baseline clinical parameters of the patients with improved LDL level and of those with non-improved LDL level. Baseline HCV RNA level and AST levels were significantly lower and CAP value was significantly higher in patients with increased LDL level. Also, baseline LSM value was significantly higher in patients with increased HDL level (Supplementary Table 2). Patient sex was not associated with LDL and HDL change after HCV eradication (p = 0.29 and p = 0.24, respectively).

## Discussion

In the current study, to elucidate whether the elimination of HCV itself could directly attenuate hepatic steatosis, we enrolled patients with significant steatosis (CAP of over 248 dB/m). As a result, hepatic steatosis was improved with the achievement of HCV eradication, as was reflected by a significant reduction in the CAP value. Of note, a factor of metabolic syndrome, the serum LDL cholesterol level, was somewhat increased. Thus, the current evidence may suggest a direct link between HCV and hepatic steatosis.

The current result seems to be inconsistent with that of a recent study reported by Ogasawara *et al*., which showed an increase in CAP after HCV eradication in CHC patients^9^. Of note, however, is the fact that the patient backgrounds at baseline were different between the two studies. Patients without significant steatosis were excluded from the current study, whereas the previous study included patients irrespective of the presence of hepatic steatosis. Thus, our study corresponds to a subgroup analysis of the previous report, focusing on the outcome of patients with hepatic steatosis.

In the natural course of CHC, a reduction in steatosis can be induced by the progression of fibrosis, a phenomenon known as “burn-out”^10^. In this previous study, hepatic steatosis might already have been diminished because of fibrosis caused by chronic HCV infection at baseline in a considerable number of patients. In such patients, it might be difficult to assess further reductions in hepatic steatosis because of HCV eradication. To eliminate the influence of liver fibrosis on steatosis at baseline as much as possible, we included only patients with significant steatosis. The induction of steatosis by HCV core protein in transgenic mice is well known^5,11,12^. The core protein that inhibits microsomal triglyceride transfer protein activity and very low density lipoprotein secretion was estimated to be a molecular mechanism of steatosis induction^13^. Furthermore, HCV infection is reportedly associated with steatohepatitis in humans^14,15^. These reports might support our finding that HCV eradication directly improved hepatic steatosis. The cancellation of core protein function may be a molecular mechanism underlying the current result. We also evaluated the baseline clinical parameters related to an improvement in hepatic steatosis after HCV eradication. A higher HDL cholesterol and a lower HbA1c level, which were strongly correlated with insulin resistance^16^, were extracted as significant factors associated with hepatic steatosis improvement. As shown in our previous study^17,18^, HCV infection and insulin resistance are closely related. Insulin resistance may play an important role as a determinant underlying hepatic steatosis improvement after HCV eradication. However, we cannot elucidate the detailed mechanism from the current study only, and further experimental studies are needed to unveil the functional relationship among HCV elimination, insulin resistance, and hepatic steatosis improvement.

In the current study, the serum LDL and HDL cholesterol levels increased significantly after HCV eradication. Similar increases in LDL cholesterol levels were reported in patients who achieved viral eradication with DAA^19^ or interferon-based treatment^20^. Our previous study showed lower serum cholesterol levels in patients with HCV infection^21^. Lipid metabolism was shown to be disrupted by chronic HCV infection^22,23^, which can be partly explained by the possible role of LDL receptors in HCV entry into cells^24,25^ or the association between HCV core protein and apolipoprotein AII^26,27^. These findings may suggest that the elevation in serum LDL or HDL levels after HCV eradication might be caused by the cancellation of the suppressive effect of chronic HCV infection on the serum lipid levels. In the current study, changes of LDL or HDL levels after HCV eradication were affected by patient clinical parameters at baseline. We cannot show the underlying structure of these findings only from the current study. Further experimental studies are needed to define the mechanism.

LSM value was significantly decreased after HCV eradication in the current study. The previous study also reported a reduction of LSM value after HCV eradication using DAA^28^. The median time between end-of-treatment and post treatment LSM measurement was only 16.1 weeks. LSM value was reported to reflect liver inflammation severity as well as fibrosis stage^29^. LSM reduction after HCV eradication may reflect a true regression of fibrosis or rather decrease in inflammation and related hepatic edema.

A major limitation of the current study is its retrospective study design and small sample size. Because of this retrospective study design, the intervals between CAP measurements were not constant. Further prospectively designed studies are needed to confirm our findings. Second, recommendations for anti-HCV treatment were based on the conditions of the patients, including age, disease severity, and the probability of SVR. Thus, the patients included in the current study may not reflect the entire natural history of chronic hepatitis C. And finally, absence of histological finding should be mentioned as limitation. While CAP measurement was reported to provide a standardized non-invasive measure of hepatic steatosis, CAP value was shown to be affected by several clinical factors (e.g. BMI or presence of diabetes)^8^.

In conclusion, we have observed a decrease in CAP after HCV eradication with DAA treatment in CHC patients with significant hepatic steatosis. The cancellation of the viral effect caused by HCV eradication is a possible underlying cause of the current findings.

## Electronic supplementary material

Supplementary tables
