# Supplementary material for: Eradication of hepatitis C virus is associated with the attenuation of steatosis as evaluated using a controlled attenuation parameter
Source: Sci Rep. 2018 May 18;8:7845. doi: 10.1038/s41598-018-26293-9 (PMC5959884; doi:10.1038/s41598-018-26293-9)
Supplement: Supplementary file 1 — Supplementary tables [file 41598_2018_26293_MOESM1_ESM.pdf]

Eradication of hepatitis C virus is associated with the attenuation of steatosis as evaluated using a controlled attenuation parameter

Kohei Shimizu[1*], Yoko Soroida[1*], Masaya Sato[1, 2], Hiromi Hikita[1], Tamaki Kobayashi[1], Momoe Endo[1], Mamiko Sato[1], Hiroaki Gotoh[1], Tomomi Iwai[1], Ryosuke Tateishi[2], Kazuhiko Koike[2], Yutaka Yatomi[1] and Hitoshi Ikeda[1,2].

[1]Department of Clinical Laboratory Medicine, and [2]Department of Gastroenterology, Graduate School of Medicine, The University of Tokyo.

*These authors equally contributed to this study.

Running Head

Hepatic steatosis alteration after HCV eradication

Corresponding Author:

Masaya Sato, M.D., Ph.D.

Department of Clinical Laboratory Medicine,

Graduate School of Medicine, The University of Tokyo

7-3-1 Hongo, Bumkyo-ku, Tokyo 113-8655, Japan

Tel +81-3-3815-5411

FAX + 81-3-3814-0021

e-mail: masayasato0407@gmail.com

Word count (Text): 1727 words

Tables: 3

Figure: 1

Conflict of interest

None of the authors have any conflicts of interest

Supplementary Table 1. Baseline clinical parameters in patients with and those without improved (decreased) LDL level

Parameters	Values		*P* -value
	Decreased	Increased	
age	49 (31-49)	68 (55-72)	0.492
Body mass index	26.1 (20.7-26.1)	22.9 (21.4-25.1)	0.696
HCV RNA (LogIU/mL)	6.9 (6.9-6.9)	6.3 (5.6-6.5)	<0.001
LSM (kPa)	16.4 (11.8-16.4)	7.5(5.4-11.6)	0.356
CAP (dB/m)	268 (264-268)	295 (257-330)	0.019
AST (U/L)	78 (73-78)	38.5 (29-54)	0.014
ALT (U/L)	79 (74-79)	33 (26-61)	0.057
γ-GTP (U/L)	81 (36-81)	29 (18-51)	0.132
Platelet Count (x10^4/μL)	19.1 (8.3-19.1)	18.3 (12.4-20.4)	0.800
Albumin (g/dL)	4.0 (3.8-4.0)	4.2 (3.8-4.5)	0.520
Total Cholesterol (mg/dL)	172 (166-172)	180 (147-200)	0.979
HDL Cholesterol (mg/dL)	55 (48-55)	53 (47-67)	0.787
LDL Cholesterol (mg/dL)	93 (92-93)	107 (77-129)	0.679
HbA1c	7.1 (7.1-7.1)	6.1 (5.5-7.1)	0.653

*Data were expressed as the median values (1st-3rd quartiles).

Supplementary Table 2. Baseline clinical parameters in patients with and those without improved (increased) HDL level

Parameters	Values		*P* -value
	Decreased	Increased	
age	68.5 (64-75)	65 (61-73)	0.434
Body mass index	22.8 (20.6-26.7)	22.2 (20.8-24.3)	0.396
HCV RNA (LogIU/mL)	6.2 (5.4-6.5)	6.5 (5.3-11.9)	0.760
LSM (kPa)	12.7 (8.6-22.0)	6.5 (5.3-11.9)	0.039
CAP (dB/m)	284 (262-315)	273 (256-307)	0.720
AST (U/L)	60 (26-73)	37 (30-50)	0.299
ALT (U/L)	59 (24-83)	35 (29-56)	0.260
γ-GTP (U/L)	33 (25-112)	23 (17-42)	0.068
Platelet Count ($x10^4/\mu L$)	18.1 (10.6-22.3)	17.4 (12.8-23.0)	0.976
Albumin (g/dL)	4.2 (3.8-4.6)	4.3 (3.9-4.4)	0.866
Total Cholesterol (mg/dL)	158 (119-158)	156 (147-201)	0.432
HDL Cholesterol (mg/dL)	60 (53-69)	55 (45-66)	0.308
LDL Cholesterol (mg/dL)	99 (59-127)	103 (81-137)	0.488
HbA1c	6.4 (5.5-6.4)	6.7 (5.5-7.6)	0.746

*Data were expressed as the median values (1st-3rd quartiles).
